# The Effects of Transcranial Electrical Stimulation on Human Motor Functions: A Comprehensive Review of Functional Neuroimaging Studies

**DOI:** 10.3389/fnins.2020.00744

**Published:** 2020-07-24

**Authors:** Yuanyuan Gao, Lora Cavuoto, Steven Schwaitzberg, Jack E. Norfleet, Xavier Intes, Suvranu De

**Affiliations:** ^1^Center for Modeling, Simulation and Imaging in Medicine, Rensselaer Polytechnic Institute, Troy, NY, United States; ^2^Department of Industrial and Systems Engineering, University at Buffalo, Buffalo, NY, United States; ^3^Department of Surgery, University at Buffalo, Buffalo, NY, United States; ^4^U.S. Army Combat Capabilities Development Command, Soldier Center (CCDC SC), Orlando, FL, United States; ^5^SFC Paul Ray Smith Simulation & Training Technology Center (STTC), Orlando, FL, United States; ^6^Medical Simulation Research Branch (MSRB), Orlando, FL, United States; ^7^Department of Biomedical Engineering, Rensselaer Polytechnic Institute, Troy, NY, United States

**Keywords:** transcranial electrical stimulation, transcranial direct current stimulation, transcranial alternating current stimulation, transcranial random noise current stimulation, neuroimaging, human motor skills, motor learning

## Abstract

Transcranial electrical stimulation (tES) is a promising tool to enhance human motor skills. However, the underlying physiological mechanisms are not fully understood. On the other hand, neuroimaging modalities provide powerful tools to map some of the neurophysiological biomarkers associated with tES. Here, a comprehensive review was undertaken to summarize the neuroimaging evidence of how tES affects human motor skills. A literature search has been done on the PubMed database, and 46 relative articles were selected. After reviewing these articles, we conclude that neuroimaging techniques are feasible to be coupled with tES and offer valuable information of cortical excitability, connectivity, and oscillations regarding the effects of tES on human motor behavior. The biomarkers derived from neuroimaging could also indicate the motor performance under tES conditions. This approach could advance the understanding of tES effects on motor skill and shed light on a new generation of adaptive stimulation models.

## Introduction

Electric brain stimulation has been reported as early as the mid-1800s (von Helmholtz, 1925), and since, its utility for clinical applications, including electroconvulsive therapy (Rudorfer et al., 2003), electroanesthesia (Kuzin et al., 1965; Brown, 1975), electrosleep (Feighner et al., 1973), and intraoperative neuromonitoring (Szelényi et al., 2007), has been extensively explored. However, these early works were performed using high-intensity transcranial electric stimulation (tES), which can lead to serious side effects and discomfort for the patient. Only in the last couple of decades, transcranial application of weak current has been demonstrated to impact brain excitability with physiological and behavioral consequences. The advantages of low-current tES include low cost, portability, minimal side effects (Matsumoto and Ugawa, 2017), and no conscious awareness of the stimulation (Nitsche and Paulus, 2000, 2001). Hence, tES is currently being employed in numerous research studies to better understand brain–behavior relations with applications in motor and cognitive rehabilitation (Flöel, 2014; Cappon et al., 2016), as well as cognitive enhancement (Dockery et al., 2009; Hoy et al., 2013; Kadosh, 2014).

Motor performance is essential to the daily life of human. The optimization of the motor performance is critical in numerous fields, such as motor skill rehabilitation, and skill-based training, as seen in athletics, aviation, driving, or surgery. Especially, tES has emerged as a promising tool to enhance human motor performance and motor learning. In animal studies (Yamada et al., 1962; Bindman et al., 1964), direct current has been shown to change cerebral excitability. Based on this finding from animal models, Nitsche and Paulus (2000) tested transcranial direct current stimulation (tDCS) on humans. They found that anodal stimulation could increase, whereas cathodal could decrease, motor cortical excitability. This interaction between electrical stimulation and cortical excitability gave rise to studies exploring whether and how tDCS could enhance human motor skills. Subsequent studies supported the facilitation effect of tDCS on motor performance and motor learning in both a healthy population (Buch et al., 2017) and patients with motor disorders (Flöel, 2014). Extending from tDCS, transcranial alternating current stimulation (tACS) delivers alternating current at constant frequencies. The alternating current may interfere with ongoing oscillations in the brain, thus changing motor performance. Antal et al. (2008) first tested tACS on humans with various frequencies, and only one specific frequency (10 Hz) could facilitate motor learning in humans. Another particular type of tES, transcranial random noise stimulation (tRNS), also features oscillation but at random frequencies. An initial experiment by Terney et al. (2008) showed the ability of tRNS to induce cortical excitability and enhance motor skill level.

**FIGURE 1:**
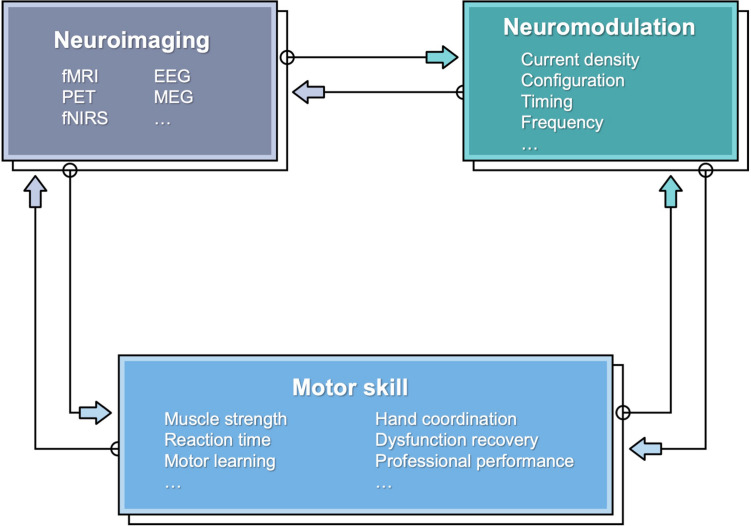
A schematic showing the relationship between motor skill, neuroimaging, and neuromodulation.

Although, still to date, and despite numerous reported studies, there is still an incomplete mechanistic understanding of the neurophysiological mechanism underlying tES effects (Liu et al., 2018). To this end, neuroimaging modalities can be leveraged to reveal neurophysiological changes (Figure 1). Indeed, modern neuroimaging methods provide means to monitor dynamical changes in the brain that could be associated with tES and thus help elucidate the relationship between neuromodulation and motor behavioral changes. The neuroimaging methods include functional magnetic resonance imaging (fMRI), diffusion tensor imaging (DTI), positron emission tomography (PET), electroencephalogram (EEG), magnetoencephalography (MEG), and functional near-infrared spectroscopy (fNIRS). Table 1 provides a comparison of the neuroimaging modalities in terms of the feasibility for combination with tES. Essential characteristics include invasiveness and temporal and spatial resolutions. Among those techniques, only PET is invasive by exposing the subjects to radiation. Functional MRI/PET has the highest spatial resolution, and EEG has the lowest. However, EEG has the highest temporal resolution, with fMRI/PET the lowest. All of the modalities could work concurrently with tES under appropriate settings. EEG and fNIRS are feasible to couple with more motor skills because of their high portability.

**TABLE 1:** Comparison between different imaging modalities for feasibility with tES.

Imaging modalities	fMRI	PET	EEG	MEG	fNIRS
Invasive	No	Yes	No	No	No
Temporal resolution	Low	Low	High	High	Medium
Spatial resolution	High	High	Low	Low (larger source location accuracy than EEG)	Medium
Online feasibility with tES	Yes (mildly affected when tES equipment is in the scan room)	Yes	Yes (signal underneath the electrode cannot be directly measured)	Yes	Yes
Portability	No	No	Yes	No	Yes
Cost	High	High	Low	High	Low to medium

Herein, we provide a review of contemporary studies that focuses on exploring the underlying mechanism of tES effects on motor function through neuroimaging-detected changes induced by tES. We first summarized the neurophysiological mechanics of tES and identified the role of neuroimaging techniques in this field. We also reviewed the tES effects under resting state before we went on to motor function–related studies. We further organized the studies mainly by imaging modality: fMRI, PET, DTI, EEG, MEG, and fNIRS. In the end, we discussed and concluded the current state of this field and future directions after the comprehensive review.

## Neurophysiological Mechanics of tEs

The modulation effects of tES on cognitive and motor functions could be understood from different mechanistic levels (Yavari et al., 2017). On the neurochemical level, neurotransmitters associated with cognitive functions [glutamatergic (*N*-methyl-D-aspartate) (Liebetanz et al., 2002; Nitsche et al., 2003, 2004a,b) and γ-aminobutyric acid–ergic receptors (Nitsche et al., 2004c)] relate to tES long-term effects; on the neuroelectrical level, cortical neuronal excitability increased/decreased under anodal/cathodal tDCS (Nitsche and Paulus, 2000, 2001); on the brain oscillatory level, tES modifies brain waves and their synchronizations (Keeser et al., 2011b; Jacobson et al., 2012; Herrmann et al., 2013).

Functional neuroimaging contributes to understanding tES mechanisms on neuroelectrical level and the brain oscillatory level, as an essential tool. Blood oxygen level–dependent (BOLD) fMRI, PET, and fNIRS measure blood flow and metabolic rate inferring the neuronal excitability changes; EEG/MEG enables analysis on brain oscillatory properties; BOLD fMRI, DTI, EEG, and fNIRS offer us neural connectivity information. The neuroimaging methods do not stand alone but support each other and could be linked back to neurochemical level analysis (Hunter et al., 2015). It is also of special interest whether neuroimaging biomarkers could indicate motor function change under tES. Thus, we will summarize and discuss the knowledge derived from these functional neuroimaging studies in the following sections.

## Neuroimaging Studies of tEs Under Resting State

Before we reviewed the effects of tES on motor functions with neuroimaging evidences, we summarized its effects under resting state as the intrinsic effects.

Under resting state, tDCS enhances cortical activation level in BOLD fMRI (Kwon et al., 2008), tracer-labeled fMRI-ASL (arterial spin labeling) (Zheng et al., 2011), and fNIRS studies (Yaqub et al., 2018). tDCS also strengthened cortical connectivity (Keeser et al., 2011a; Polanía et al., 2011b, 2012a,b; Peña-Gómez et al., 2012). The connectivity of functionally and topologically close areas increased after tDCS (Polanía et al., 2011b), both under and distant from the electrodes (Keeser et al., 2011a), including corticostriatal and thalamocortical circuits (Polanía et al., 2012b). Anodal tDCS increases task-related connectivity and depresses anti–related default-mode network (Peña-Gómez et al., 2012) [cathodal decreased it (Amadi et al., 2014)], which may explain the facilitative effects on cognitive and motor functions. The resting-state connectivity could also be increased by tACS with sophisticated selected parameters (Bächinger et al., 2017). The efficiency of changing the connectivity correlates to the baseline connectivity level (Polanía et al., 2012a; Bächinger et al., 2017). In contrary to the enhancement effect of tDCS on connectivity, some studies showed that tDCS decoupled local and the interhemispheric connectivity (Sehm et al., 2013; Yan et al., 2015).

In summary, both cortical activation and connectivity were enhanced by tES under resting state. Based on this understanding, we further reviewed the neuroimaging studies related to motor performance in the following sections.

## Review Criteria

The review focused on studies utilizing neuroimaging tools to explore the neuromodulation effects of tES on human motor performance or motor learning. The search was carried out in April 2020 based on the PubMed database. The keywords used during the search included the following: “transcranial electrical stimulation (tES)” OR “transcranial direct current stimulation (tDCS)” OR “transcranial alternating current stimulation (tACS)” OR “transcranial random noise stimulation (tRNS)” AND “neuroimaging” OR “functional magnetic resonance imaging (fMRI)” OR “positron-emission tomography (PET)” OR “electroencephalogram (EEG)” OR “Magnetoencephalography (MEG)” OR “functional near-infrared spectroscopy (fNIRS)” AND “motor.” The articles were filtered by article types: “Clinical trial” OR “Journal article,” species: “Human study” and languages: “English,” resulting in 163 articles. The reference lists of the resulting articles were scanned to identify further relevant studies, which resulted in 120 additional results. After removing duplicate results, the search resulted in 242 related articles.

**FIGURE 2:**
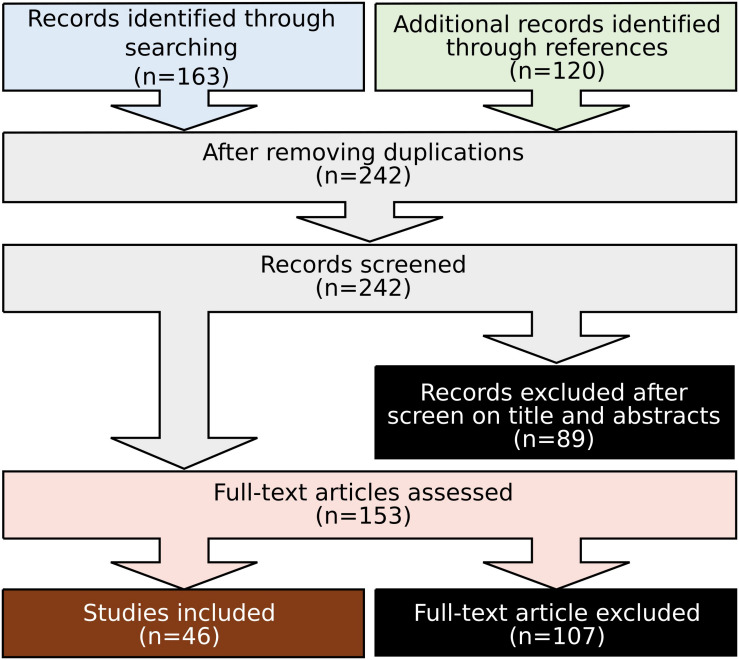
Flow diagram of study selection.

After searching, we employed the following inclusion and exclusion criteria to select relevant works for detailed review. Only studies that investigated how neuromodulation affects motor performance or motor learning revealed by neuroimaging techniques were included. All studies investigating the resting state or intrinsic neuronal response were excluded. Studies with only motor imagery were also excluded. Investigations of cognitive paradigms involving motor responses (e.g., “Go No Go”) are beyond the scope of this review and were excluded. Such an approach led to a total of 46 publications fulfilling all criteria (Figure 2). From each article, we extracted the information including stimulation parameters (electrode position, current intensity, current density, stimulation durance, frequency, and application days), neuroimaging parameters (electrode position for EEG/MEG, optode position for fNIRS), motor task involved, and reported observation (Tables 2–4).

**TABLE 2:** Transcranial electrical stimulation effect detected by fMRI/PET.

References	Electrode position (Anode– cathode; “i” = ipsilateral; “c” = contralateral)							Stimulation parameter					Stimulation-affected area						Motor task	Effects	Cohort
						
	iM1-cSO	iM1-shoulder	iM1-cM1	cSO-iM1	iSO-cM1	cM1-iM1	Cz-FCz	I (mA)	J (mA/cm^2^)	t (min)	f (Hz)	Days	SMA	M1	PFC	Pre-motor	S1	Insula, frontoparietal			
***fMRI***																					
Baudewig et al. (2001)	•^1^			•^2^				1	0.04	5	0	1	•^2^						Hand grasp–release	Short term (0–5 min; 15–20 min)	Healthy
Chaieb et al. (2009)	•							1	0.06	4	0–640	1		•					Index–thumb grasp–release	Short term (0–40 min)	Healthy
Jang et al. (2009)	•							1	0.03	20	0	1	•	•		•			Hand grasp–release	Short term (0–2 min)	Healthy
Stagg et al. (2009)	•			•				1	0.03	10	0	1	•	•		•			SFTT	Short term (1,5,10,15,20 min)	Healthy
Lindenberg et al. (2010)			•					1.5	0.09	30	0	5		•		•			Wrist/elbow extension and flexion	Long term (3 days; 7 days)	Stroke
Antal et al. (2011)	•			•				1	0.03	0.33	0	1	•						Index–thumb grasp–release	Online	Healthy
Nair et al. (2011)				•				1	•	30	0	5		•					Wrist/elbow extension and flexion	Long term (7 days)	Stroke
Kwon and Jang (2011)	•							1	0.03	2	0	1		•					Hand grasp–release	Online	Healthy
Stagg et al. (2012)	•				•			1	0.03	10	0	1	•	•		•			Response time	Short term (0 s)	Stroke
Kim et al. (2012)	•							2	0.08	15	0	4	•	•					Toe flexion	Short term (0 s)	Healthy
Saiote et al. (2013a)	•							1	0.03	10	0.1–100; 101–640	1			•				Force tracking	Online and short term (20 min)	Healthy
Lefebvre et al. (2015)			•					1	0.03	30	0	1		•		•			Drawing	Online, short term (0 s, 30 min, 60 min), and long term (1 week)	Stroke
Allman et al. (2016)	•							1	0.03	20	0	9		•		•			WMFT and FMA	Short term (0 s) and long term (1 week, 1 month, 3 months)	Stroke
Moisa et al. (2016)		•						1	0.03	20	20;70	1		•	•				Force tracking	Online	Healthy
Cabral-Calderin et al. (2016)							•	1.5	0.09	0.2	16^1^; 80^2^	1					•	•^1^	Sequential finger opposition	Online	Healthy
Waters et al. (2017)	•		•			•		2	0.06	25	0	4		•			•		SFTT	Online, short term (0–35 min) and long term (1–2 days)	Healthy
Lefebvre et al. (2017)	•							1	0.03	30	0	1		•		•			Drawing	Online, short term (0 s, 30 min, 60 min), and long term (1 week)	Stroke
Liu et al. (2019)		•						1;2	0.07; 0.14	20	0	10			•				Rowing training	Long term (2 weeks)	Athletes
Lee et al. (2018)				•				2	0.06	20	0	10	Interhemispheric connectivity						FMA	Long term (2 months)	Stroke
Lee et al. (2019)	•							2	0.06	20		10	Balance of M1 intrahemispheric connectivity, enhanced in responders who improved their FMA scores after tDCS.						FMA	Long term (2 months)	Stroke
***DTI***																					
Lindenberg et al. (2012)			•					1.5	•	30	0	5	More the ipsilesional FA profiles of patients resembled those of healthy controls, the greater their functional improvement.						FMA	Long term (3, 7 days)	Stroke
Lindenberg et al. (2013)	•^1^		•^2^					1	0.03	30	0	1	Higher FA values of transcallosal motor tracts^2^.						Choice reaction finger tapping	Online	Older adults
Zheng and Schlaug (2015)			•					1.5	0.09	30	0	10	Significant increases in FA were found in the ipsilesional aMF in the treated group.						FMA	Short term (after intervention)	Stroke
Lindenberg et al. (2016)	•^1^		•^2^					1	0.03	30	0	1	Responders to dual-tDCS were characterized by stronger transcallosal connections between bilateral M1^2^.						Choice reaction finger tapping	Online	Healthy
Allman et al. (2016)	•							1	0.03	20	0	9	Higher FA values correlated to performance enhancement.						WMFT and FMA	Short term (0 s) and long term (1 week, 1 month, 3 months)	Stroke
***PET***																					
Paquette et al. (2011)	•							2	0.08	4	0	1	rCBF increased under anodal and decreased under cathodal tDCS.						Sequential finger opposition	Online	Healthy
Lang et al. (2005)				•				1	0.03	10	0	1	rCBF increased under anodal and decreased under cathodal.						Simple tapping task and sequential tapping task.	Short term (after intervention)	Healthy

*SFTT, serial finger tapping task; WMFT, Wolf motor function test; FMA, Fugl–Meyer assessment scale; FA, fractional anisotropy; rCBF, regional cerebral blood flow; sequential finger opposition: sequential tapping of the index, middle, ring, and fourth finger against the thumb; simple tapping: subjects tapped their right index finger as many times as possible during a 10-second interval; sequential tapping task: subjects were asked to repeat an ascending sequence (index, middle, ring, little finger) as quickly as possible for 10 s. Superscript numbers link the settings and the findings accordingly. In “Effects” section, “online” refers to motor changes happened during the tES, “short term” refers to changes in a short time (<1 day) after the tES, “long term” refers to changes in a long time (>1 day) after tES.*

**TABLE 3:** Transcranial electrical stimulation effect detected by EEG/MEG.

Reference	tES Electrode position (anode–cathode and “i” = ipsilateral; “c” = contralateral)	EEG Electrode position	Stimulation parameter					Result	Motor task	Effects	Cohort
			I (mA)	J (mA/cm^2^)	t (min)	f (Hz)	Days				
**EEG**											
Antal et al. (2008)	iM1-cSO	Cz, C3, C4	0.4	0.03	5	1;10;15;30;45	1	No significant effect	SRTT	Short term (5 min)	Healthy
Terney et al. (2008)	iM1-cSO	Cz, C3, C4	1	0.06	10	0–640	1	No significant effect	SRTT	Online and short term (0–60 min)	Healthy
Polanía et al. (2011a)	iM1-cSO	62 channels	1	0.06	10	0	1	FC increased in PMd, M1 in 60–90 Hz	Index–thumb grasp–release	Short term (after intervention)	Healthy
Notturno et al. (2014)	iM1-cSO cSO-iM1	32 channels	1	0.03	20	0	1	An increment of low alpha band ERD in bilateral central, frontal areas and in the left inferior parietal region; An increment of beta ERD in frontocentral and parieto-occipital regions	Sequential finger opposition	Short term (after intervention)	Healthy
Dutta et al. (2014)	iM1-cSO	F3, F4, P3, P4	1.66	0.53	15	0	1	Decreased the slope of post-tDCS SCP	Ankle dorsiflexions	Online and short term (10 min)	Healthy
Marquez et al. (2015)	iM1-cSO	64 channels	1	0.03	20	0	1	No significant effect	JTT; muscle strength	Short term (after intervention)	Older adults
Choe et al. (2016)	M1 redial fashion^1^ DLPFC radial fashion^2^	32 channels	2	0.04	60	0	1	Parietal alpha activity increased^1^; midline frontal theta activity increased^2^	Pilot	Online	Healthy
Berger et al. (2018)	Parietal	F3, Fz, F4, Cz, Pz	1	0.32	20	10;20	1	Parietal alpha activity increased	Bimanual	Short term (0 s, 30 min) and long term (1 day)	Healthy
Schoellmann et al. (2019)	iM1-cSO	25 channels	1	0.03	20	0	1	Reduced coherence from 22 to 27 Hz over the left sensorimotor and right frontotemporal area	Grip task	Short term (3 and 29 min)	Parkinson disease and healthy
Del Felice et al. (2019)	EEG power spectral difference locations	32 channels	1– 2	0.03– 0.06	30	4;30;0-100	10	A reduction of beta rhythm offline over right sensorimotor area and left parietal area and follow-up over right sensorimotor area and left frontal area	Physical therapy	Short term (after intervention) and long term (4 weeks)	Parkinson disease
Berntsen et al. (2019)	P3-FP2; between F5 and F7 – FP2; C3-FP2	64 channels	1	0.11	20	IAF	1	Enhanced the motor performance after prefrontal IAF-tACS; A reduction in low beta ERD	Sequential hand motion	Short term (after intervention)	Healthy

*In “Effects” section, “online” refers to motor changes happened during the tES, “short term” refers to changes in a short time (<1 day) after the tES, “long term” refers to changes in a long time (>1 day) after tES. FC, functional connectivity; ERD, event-related desynchronization; SCP, slow cortical potentials that are defined as those positive or negative polarizations of the EEG that last from 300 ms to several seconds before electromyography onset with magnitudes up to 50 μV; IAF, individual alpha frequency, individual’s maximum amplitude between 8 and 12 Hz over parietal and occipital electrodes; CMC, corticomuscular coupling; SRTT, serial reaction time task; JTT, Jebsen Taylor hand function test. Sequential finger opposition: sequential tapping of the index, middle, ring, and fourth finger against the thumb. Superscript numbers link the settings and the findings accordingly.*

**TABLE 4:** tES effect detected by fNIRS.

Reference	Electrode position (anode–cathode and “i” = ipsilateral; “c” = contralateral)	Optode position	Stimulation parameter					Measurement			Motor task	Effects	Cohort
			I (mA)	J (mA/cm^2^)	t (min)	f (Hz)	Days	HbO	HbR	connectivity			
Khan et al. (2013)	iM1-cM1 cM1-iM1	PMC, M1, S1, PPC	2	0.08	15	0	1	•		•	Muscle contraction	Online and short term (after intervention)	Healthy
Khan et al. (2015)	iM1-cM1 20 combinations between PMC, M1 and SO	PMC, M1, S1, PPC	0.5	0.02	0.67	0	1			•	Force tracking	Short term (5 min)	Healthy and Stroke
Angius et al. (2016)	iM1-cSO iM1-shoulder	PFC	2	0.17	10	0	1	∘	∘		Muscle contraction	Short term (after intervention)	Active males
Muthalib et al. (2016)	M1-radial	Sensorimotor	2	0.67	10	0	1		•		Sequential finger opposition	Online and short term (3 min)	Healthy
Choe et al. (2016)	M1-radial DLPFC-radial	Left M1, right DLPFC	2	0.04	60	0	4	∘	∘		Pilot training	Online	Healthy
Radel et al. (2017)	PFC region	Right lateral PFC and the right M1	2	0.32	20	0	1	•			Muscle contraction	Online and short term (after intervention)	Healthy
Berger et al. (2018)	Parietal	M1	1	0.3	20	0	1	•			Bimanual joysticks	Short term (0 s, 30 min) and long term (1 day)	Healthy
Ciechanski et al. (2019)	iM1-cSO	32 channels	1	1.27	20	0	1	Enhanced the motor performance; an increment in beta power			Surgical task	Online and short term (after intervention)	Healthy
**MEG**													
Krause et al. (2014)	iM1-cSO	306 channels	1	0.03	15	10;20	1	Attenuated beta band CMC during isometric contraction after 20-Hz tACS			Forearm isometric contraction	Short term (after intervention)	Parkinson disease and healthy
Hanley et al. (2016)	Oz - Cz; C3 - Fp2	275 channels	1	0.03	10	0	1	Significantly modulated motor-evoked responses			Right index finger abduction	Online and short term (after intervention)	Healthy
Sugata et al. (2018)	iM1-cSO	306 channels	1	0.03	10	10;20;70	1	A significant increase in beta-band power after 70-Hz tACS			Finger tapping	Short term (after intervention)	Healthy

*“∘” means no significant change observed. PMC, premotor cortex; S, primary somatosensory cortex; PPC, posterior parietal cortex. Sequential finger opposition: sequential tapping of the index, middle, ring, and fourth finger against the thumb. In “Effects” section, “online” refers to motor changes happened during the tES, “short term” refers to changes in a short time (<1 day) after the tES, “long term” refers to changes in a long time (>1 day) after tES.*

## Bold fMRI

Because of its high spatial resolution, fMRI can detect full brain functional activation induced by tES. A comprehensive overview of the combination of fMRI with tES has been proposed recently in two review articles (Turi et al., 2012; Saiote et al., 2013b). Turi et al. (2012) discussed how to combine tES and fMRI technically and summarized the results “online” and “offline” accordingly (with “online” referring to fMRI scan simultaneously with tES, “offline” referring to fMRI scan after tES), whereas Saiote et al. (2013b) summarized motor task–related activation by tES detected by fMRI in one section. Herein, we further restrict the overview of this field to motor behavioral changes in correlation with fMRI results (Table 2).

### tDCS

#### Offline

**FIGURE 3:**
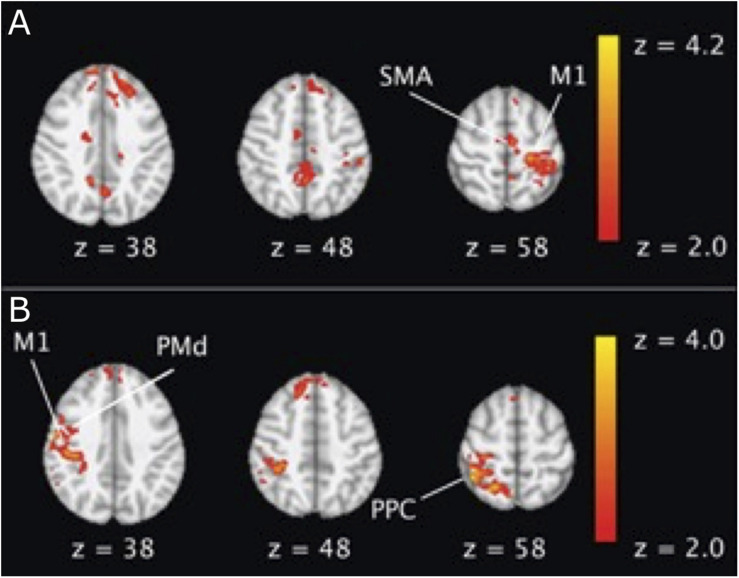
An example of BOLD fMRI difference before and after anodal tDCS compared with the difference before and after cathodal tDCS. **(A)** Right anodal tDCS increased the activation in SMA and ipsilateral M1; **(B)** right cathodal tDCS increased the activation in contralateral M1, dorsal premotor cortex (PMd), and posterior parietal cortex (PPC) region. The figure is adapted with permission from Stagg et al. (2009).

The offline effect, that is, the cortical activation before and after stimulation, could be measured by fMRI. In line with the consensus that anodal tDCS increases the excitability, while cathodal decreases it, Baudewig et al. (2001) observed a global decrease in the number of activated pixels after cathodal tDCS and an increase after anodal. The activation of the cortex under the anodal electrode increased in the majority of articles (Jang et al., 2009; Stagg et al., 2009, 2012; Lindenberg et al., 2010; Allman et al., 2016). However, the cortical areas far away from the electrodes increased their excitability due to the stimulation, such as supplementary motor area (SMA) (Baudewig et al., 2001; Jang et al., 2009; Stagg et al., 2009, 2012), premotor area (Jang et al., 2009; Stagg et al., 2009, 2012; Lindenberg et al., 2010; Allman et al., 2016), and parietal area (Stagg et al., 2009; Figure 3). Lefebvre et al. (2015) obtained fMRI images during a retention test 1 week after motor training. They observed that tDCS activated the premotor area exclusively, whereas sham activated a widespread region including the bilateral primary motor cortex (M1), the primary somatosensory cortex (S1), and the parietal cortex. The individual contribution analysis also showed more widespread interindividual activation in sham. This observation indicated that the lasting behavioral enhancement associates with more efficient recruitment of the motor skill learning network, reflected by focused activation. Application of tDCS over multiple days also led to increased M1 region activation in individuals with stroke (Lindenberg et al., 2010) and those who are healthy (Waters et al., 2017), as well as SMA activation in healthy adults (Kim et al., 2012).

#### Online

**FIGURE 4:**
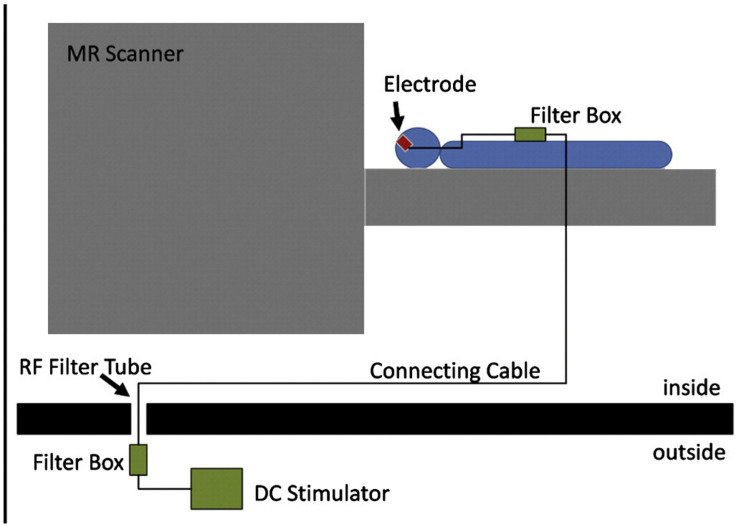
Experimental setup for concurrent tDCS and fMRI. The figure is adapted with permission from Antal et al. (2011).

Concurrent fMRI with tDCS and motor performance can provide information about the real-time effects of tDCS. Antal et al. (2011) explored the feasibility of concurrent fMRI and tDCS using specially designed fMRI-compatible electrodes, with a 5.6 kΩ resistance to avoid heating due to induction voltages from radiofrequency pulses (setup illustrated in Figure 4). The signal-to-noise ratio of the fMRI images with the electrodes was reduced by only 3 to 8% (Antal et al., 2011). Based on the feasibility, several studies have been conducted on motor performance by concurrent fMRI and tDCS. Similar to offline effects, online anodal tDCS during motor task performance enhanced cortical activation in the M1 region (under the electrodes) (Kwon and Jang, 2011), whereas cathodal decreased it (Nair et al., 2011). In contrast to offline tDCS, the anodal online tDCS decreased the activation in SMA (Antal et al., 2011), indicating different effects of online and offline tES.

#### Connectivity

Functional connectivity could be derived from the fMRI technique by defining regional cross-correlations of the time courses of BOLD signal changes. The functional connectivity between the contralateral (right) M1 and premotor cortex (PMd) was observed to increase after cathodal stimulation when contrasted with sham (Stagg et al., 2009). Relative increases in functional connectivity were also seen bilaterally in the dentate nuclei within the cerebellum (Stagg et al., 2009). Similarly, in a study of individuals with stroke (Lefebvre et al., 2017), 1 week after dual-tDCS, the most active functional connection was found between the M1 and PMd of the damaged hemisphere, based on seed-based analyses. In Liu et al. (2019), a significantly increased bilateral hemispherical connection of the middle temporal gyrus, precentral gyrus, and superior frontal gyrus was found in the high-stimulation group (2 mA) 1 weeks after 10 days of repeated tDCS followed (Zuo et al., 2010). Lee et al. (2018) tested the effect of additional cathodal tDCS to conventional 10-Hz repetitive transcranial magnetic stimulation. The additional tDCS noticeably increased connectivity and network efficiency in stroke patients. Their follow-up study (Lee et al., 2019) indicated that some of the stroke patients (“responders”) improved their motor ability, whereas others (“non-responders”) did not. By the graph theory functional connectivity analysis, they revealed that M1 tDCS could restore the noticeable imbalance of intrahemispheric connectivity between affected and unaffected hemispheres in responders. Further analysis showed that responders are featured by greater interhemispheric connectivity and higher efficiency of the motor network than the non-responders.

### tACS

The ability to affect remote sites has also been observed in tACS. Moisa et al. (2016) showed that gamma tACS induced motor performance enhancement and correlated with changed BOLD activity in the stimulated M1. Moreover, these facilitatory effects are accompanied by decreased brain activity in a remote brain region, indicating that tACS could affect the areas that are connected and integrated functionally with the stimulated regions. Similarly, in Cabral-Calderin et al. (2016), during a finger-tapping task, tACS only increased the activity in distant areas (the insula, frontoparietal, and occipital regions) from motor-related areas.

### tRNS

**FIGURE 5:**
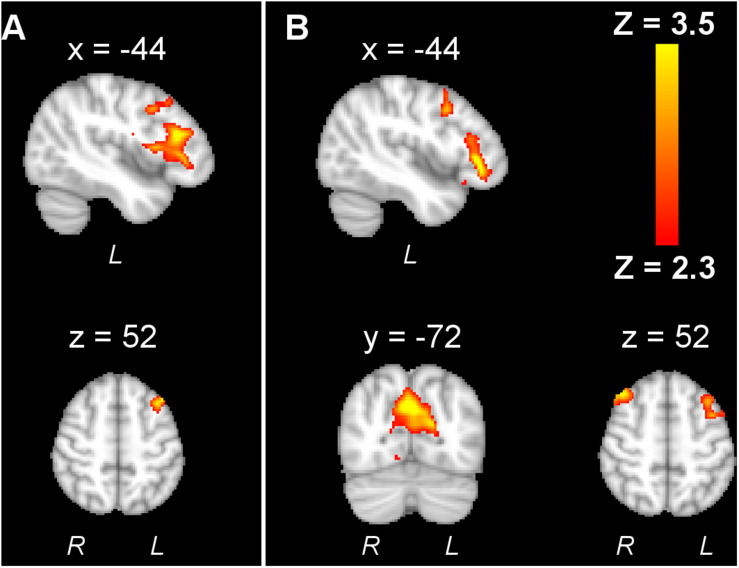
An example adapted from Saiote et al. (2013a) showing that tRNS decreased activation in the left frontal cortex, and high-frequency tRNS further decreased right frontal cortex and precuneus activation. The reproduction of this figure is under the terms of the Creative Commons Attribution License.

Unlike tDCS and tACS, tRNS has led to deactivation effects. Chaieb et al. (2009) showed that the motor task–activated pixels decreased by 17% in the hand area after tRNS. Although surprising at first glance, the authors tried to explain the phenomenon through the homeostatic response and stochastic resonance theory. Saiote et al. (2013a) compared the BOLD by high-frequency tRNS (hf-tRNS) to low-frequency tRNS. Interestingly, the prefrontal cortex (PFC) and precuneus, which is located deep in the medial longitudinal fissure between the two cerebral hemispheres, were deactivated by tRNS (Figure 5). The authors explained that an increase in neuronal synchronization by hf-tRNS would lead to greater efficiency and a consequent decrease in activity (Chaieb et al., 2009). The different observations between tDCS and tRNS activation effects call for further exploration into the mechanism of tRNS.

## DTI

Diffusion tensor imaging is one of the diffusion-weighted MRI methods. Diffusion tensor imaging maps and characterizes the three-dimensional diffusion of water. The principal diffusion eigenvector is assumed to be parallel to the tract orientation in homogeneous white matter. Thus, this technique detects the changes in tracts and connections at the cellular and microstructural levels. The most common measurement of DTI is fractional anisotropy (FA). It indicates fiber alignment, myelination, and overall fiber integrity. Other measures include radial and axial diffusivities, which provide more specific information about the diffusion tensor.

For motor function studies, DTI stands out compared to other neuroimaging techniques, by detecting the changes of the motor tracts induced by tES. Lindenberg et al. (2012) and Allman et al. (2016) have explored the potential of DTI to predict stroke recovery. They found that motor functions were related to FA and directional diffusivities of corticospinal tracts. Also, ipsilesional FA profiles of patients were related to their functional improvement (Lindenberg et al., 2012). Their subsequent work (Zheng and Schlaug, 2015) showed increased FA in ipsilesional alternate motor fibers (aMFs) in tDCS-treated group but not in the control group. It indicates that the mechanism behind motor improvement from tDCS may be the ability to reconstruct the motor fibers in aMFs. Their other works on healthy adults, both young (Lindenberg et al., 2016) and old (Lindenberg et al., 2013), showed that dual-tDCS was characterized by higher FA values of transcallosal motor tracts.

## PET

Similar to fMRI, PET can also map the functional brain activation with relatively high spatial resolution. Positron emission tomography works by injecting a radiopharmaceutical into the body, typically [18F]-fluoro-2-deoxy-D-glucose (^18^FDG), which can be detected by a PET scanner (Cabeza and Nyberg, 1997). Thus, the PET signal is an index of the distribution of the cerebral blood flow and regional metabolic rate associated with local neuronal activity, indirectly offering the brain activity pattern (Cabeza and Nyberg, 1997). Based on the set review criteria, we identified two articles (Table 2) reporting on investigating tDCS effects via PET imaging. Both observed increased regional cerebral blood flow caused by tDCS during a sequential finger opposition task performance (Paquette et al., 2011), simple tapping task, and sequential tapping task (Lang et al., 2005).

## EEG

Electroencephalogram is a non-invasive technique to measure the electrical signal changes. Because the EEG signal is directly coupled to neuronal electrical activity, the utility of it combined with tES could offer us information about how tES changes the neuronal electrical activity. The studies combining tES and EEG have been reviewed (Miniussi et al., 2012). Here we focus on motor-related studies only and go further into the relationship between motor skills, tES, and EEG (Table 3).

### Oscillation Analysis

Frequency power analysis in EEG is a conventional way to examine the brain oscillatory properties. In the early studies by Antal et al. (2008) and Terney et al. (2008), no significant difference in EEG power was observed after either tACS (1, 10, 15, 30, and 45 Hz) or tRNS, although significant motor learning behavior difference occurred. These results may be due to the super low-density EEG montages used (only three channels). Choe et al. (2016) observed that midline frontal theta band power increased under the dorsolateral prefrontal cortex (DLPFC) tDCS, whereas parietal alpha power increased under M1 tDCS during flight tasks. Although only five electrodes were used in Berger et al. (2018), parietal alpha power increase under parietal tDCS was also observed. Del Felice et al. (2019) found a reduction of excessive beta rhythm in Parkinson disease (PD) patients after theta-tACS versus tRNS in sensorimotor, left parietal areas, and left frontal area. While in healthy participants, low beta event-related desynchronization (ERD) reduced after individual alpha frequency tACS (Berntsen et al., 2019). However, beta power increased during surgical motor tasks after tDCS (Ciechanski et al., 2019). Dutta et al. (2014) observed the slope of slow cortical potentials (SCPs) of ankle flexions decreased after tDCS. From this observation, the authors postulated that change in the slope of SCP might be related to the reaction times during a cued movement task. Neither behavioral nor EEG signal change was induced by tDCS in an older population (Marquez et al., 2015).

### Connectivity

Multichannel time-series EEG coherence matrix offers us insights into brain connectivity. Sixty-two-channel EEG was used in Polanía et al. (2011a) to investigate the impact of tDCS onto brain synchronization and topological functional organization. It revealed that tDCS increased functional connectivity within the premotor, motor, and sensorimotor areas during voluntary hand movements in the high-gamma band (60–90 Hz). Schoellmann et al. (2019) discovered a reduction of beta rhythm coherence in PD patients after tDCS. Because excessive beta synchronization in the beta range is a marker in PD patients, these findings could indicate the potential application of tDCS in alleviating PD symptoms.

## MEG

Magnetoencephalography can be used to measure neuromagnetic activity non-invasively (Soekadar et al., 2013). Although the MEG signal is highly correlated to EEG, it is nearly independent of the distorting effects of biological conduction boundaries such as the scalp compared to EEG (Okada et al., 1999). The feasibility of simultaneously recording MEG during tES was demonstrated in Soekadar et al. (2013).

Hanley et al. (2016) observed the significantly increased amplitude in motor-evoked responses by anodal stimulation. The corticomuscular coupling (CMC) is calculated as the linear relationships between electromyography and MEG signals. It quantifies the functional coupling between M1 and the contralateral peripheral muscle in the frequency domain. Krause et al. (2014) showed attenuated beta-band CMC during isometric contraction after 20-Hz tACS. Sugata et al. (2018) tested implicit finger tapping learning performance after 10-, 20-, and 70-Hz tACS and found that the capacity for motor learning increased for 70-Hz tACS. The oscillation analysis also revealed a significant increase in beta-band power after 70-Hz tACS but not in the other stimulation groups.

## fNIRS

Functional near-infrared spectroscopy is a non-invasive optical technique measuring hemodynamic changes, and it has several advantages for seamless integration with tES. First, it has a relatively higher time resolution compared to fMRI and higher spatial resolution compared to EEG. Second, fNIRS is an optical technique that does not affect the tES electrical signal, and the electrical field generated by tES does not affect the optical density data collected by fNIRS. Last, the tES electrode position close to the EEG electrode could cause the saturation of the recording electrodes, thus making the measurement of the area under the tES electrode difficult. While in the fNIRS setting, it could be easily measured by setting the tES electrode along the light path of near-infrared light. The integration of tES and fNIRS was reviewed in McKendrick et al. (2015). Here we focus on motor skills related studies with recent findings (Table 4).

Khan et al. (2013) showed that the motor cortex oxyhemoglobin (HbO) increased under the anodal tDCS during a muscle contraction task. Angius et al. (2016) demonstrated that tDCS was able to improve knee extensor ability, but no significant changes were observed in hemodynamic concentrations. Muthalib et al. (2016) observed smaller decreases in deoxyhemoglobin (HbR) due to tDCS. Ten- and 20-Hz tACS (Berger et al., 2018) and PFC location tDCS (Radel et al., 2017) have been shown to decrease the HbO.

The synchronization analysis in fNIRS data also allowed studying connectivity. Studies by Khan et al. (2013, 2015) showed that the interhemispheric connectivity increased with tDCS during a muscle contraction task.

Despite the advantages of integrating fNIRS with tES, only a few studies have been reported. The cortical excitation results vary under different experimental conditions (motor tasks, electrode locations), resulting in limited understanding of how tES affects the human motor skills revealed by fNIRS measurement.

## Suggested Neuroimaging Biomarkers for Motor Performance Under tEs

Whether neuroimaging biomarkers could predict the motor skill level improvement is drawing researchers’ attention. Blood oxygen level–dependent fMRI analysis suggested such biomarkers. For example, the precentral gyrus activation laterality index correlated with Wolf motor function test (WMFT) score changes in the real stimulation group (Pearson coefficient *r* = 0.72, *p* = 0.029), not in the sham group (Lindenberg et al., 2010). Activation in the contralesional motor region related to Fugl-Meyer assessment (FMA) score (*R*^2^ = 0.275; *p* = 0.033; one-tailed) (Nair et al., 2011). Ipsilesional activation related to response times (*r* = 0.902) (Stagg et al., 2012). A week after dual-tDCS, a correlation between the beta weights, and the learning index (*r* = 0.72, *p* = 0.04), performance index (*r* = 0.81, *p* = 0.02) was found in the ipsilesional premotor area (Lefebvre et al., 2015). In an fNIRS study, Choe et al. (2016) showed that the performance improvement in pilot training due to tDCS is related to DLPFC and M1 region HbO. In EEG/MEG studies, strong correlations were observed between changes in beta band power (Sugata et al., 2018), alpha and gamma band power (Ciechanski et al., 2019), and motor learning performance.

## Discussion and Conclusion

### Summary of the Review

The effect of tES on behavioral motor skills has been documented in multiple studies (Buch et al., 2017). However, the understanding of the mechanism is still limited. Neuroimaging is well-positioned to enable elucidating the connection between tES stimulation and motor behavior changes. Here we reviewed the studies that have combined tES and neuroimaging methods to investigate human motor performance or motor learning ability. Overall, the collective findings summarized herein support the feasibility of monitoring various tES neurophysiological effects via various neuroimaging modalities.

**FIGURE 6:**
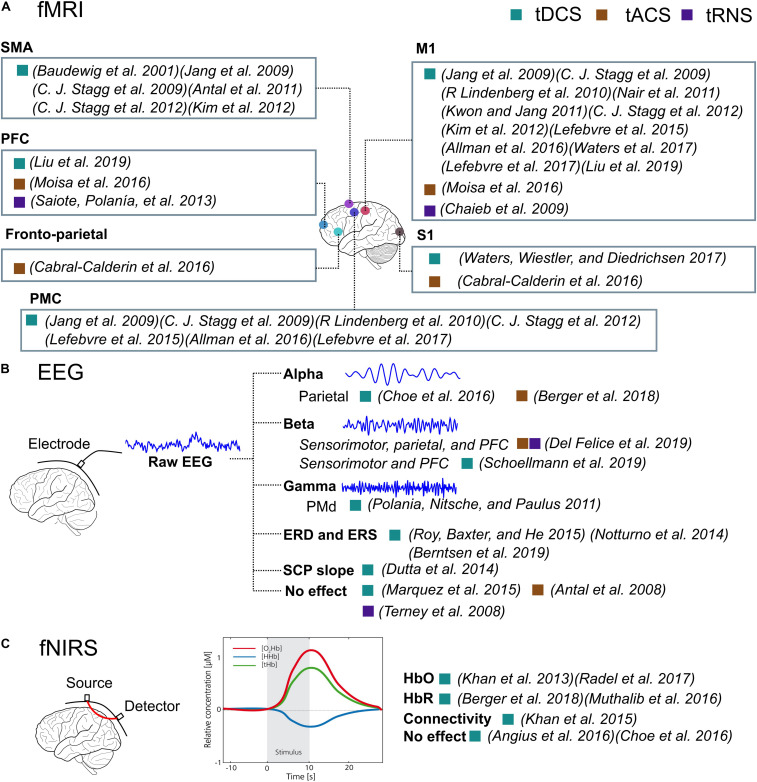
The illustration of the effect of tES detected by fMRI, EEG, and fNIRS. The filled rectangular dots represent the stimulation type, and the filled circles represent cortical sites. **(A)** The cortical regions are illustrated where the functional activations were significantly affected by tES revealed by fMRI imaging. **(B)** The representative signals of raw EEG signal, alpha band, beta band, and gamma band signals and how they are affected by tES in papers. **(C)** An illustration of fNIRS and which measurement, HbO, HbR, or connectivity, is affected by tES. An illustration of the fNIRS signal is also present.

The results of the fMRI and PET studies showed increased functional activation following anodal tDCS and tACS, compared to before the stimulation, or increased online compared to offline. This effect was present not only at the sites under the electrodes but also the remote areas (Figure 6A), showing a spread effect of tES and the inner-cortical functional connectivity. On the other hand, tRNS resulted in decreased activation in several studies, indicating a different mechanism from tDCS or tACS. Other than fMRI and PET, the techniques of EEG, MEG, and fNIRS also showed increased excitability and connectivity after tES compared to before tES, or increased online compared to offline, or compared to sham (Figures 6B,C). The measured outputs are time-series data, from which spectral power, connectivity could be derived. This is especially useful when studying tACS and tRNS whose mechanism is interacting with the ongoing cortical oscillation. Other time-series data-based measurements such as ERD and SCP could be derived. Transcranial electrical stimulation decreased the slope of SCP and increased ERD in specific bands. Both simple and complex tasks were used. For simple tasks, power, connectivity, ERD, SCP, CMC, motor-evoked response, and HbO and HbR levels were affected. For complex motor tasks, parietal alpha activity increased in pilot training task (Choe et al., 2016) and a bimanual joystick control task (Berger et al., 2018). The HbO level has also been reported to be altered in the bimanual joystick control task (Berger et al., 2018), but not in the pilot training task (Choe et al., 2016). The information for the complex tasks is still limited from this review.

On the technical side, the primary stimulation location across studies was the M1 region, which plays an essential role in voluntary motor control (Sanes and Donoghue, 2000). Other locations of targets include PFC, DLPFC, and parietal cortex. In a pilot training study (Choe et al., 2016), DLPFC tDCS had the same behavior effects except it reduced the variability in online learning. Khan et al. (2015) tested 20 combinations of electrode locations. Among them, bilateral PMd yielded more accurate and faster performance, but each individual reacted differently. The current intensity ranged from 1 to 2 mA, and the current density ranged from 0.03 to 0.14 mA/cm^2^. One study (Liu et al., 2019) compared high (2 mA) and low intensity (1 mA) under the same experimental settings and observed higher brain activation in high stimulation. Across the studies reviewed, the stimulation duration was up to 30 min. The number of sessions is up to 10. Researchers tend to report long-term retention effects with longer stimulation duration and more sessions, such as (Nair et al., 2011; Lindenberg et al., 2012; Allman et al., 2016; Waters et al., 2017; Lee et al., 2018, 2019; Liu et al., 2019). The long-term effects were reported up to 2 months after the stimulation (Lee et al., 2019).

Most studies adopted basic motor tasks, such as hand grasp–release or wrist/elbow flexion and extension. These tasks were used to elicit motor execution functional activation in the motor cortex and investigate how tES affects the activation. As they require minimal body motion, they allowed for completion within the confines of the fMRI and PET scanners. Some studies evaluated complex motor tasks that involve higher motor ability and could be used to quantify changes in motor ability attributable to tES. These motor tasks include (i) motor learning: serial reaction time task, and serial finger tapping task; (ii) hand–eye coordination: force tracking, and drawing; (iii) dexterity: WMFT and FMA test.

Some studies focused on patients with neurological disorders (Tables 2–4). For stroke patients, tDCS increased their hand–eye coordination (Lefebvre et al., 2015, 2017) and dexterity (Lindenberg et al., 2010, 2012; Nair et al., 2011; Khan et al., 2015; Zheng and Schlaug, 2015; Allman et al., 2016; Lee et al., 2018, 2019), which could last for 1 week to 2 months. Two studies (Del Felice et al., 2019; Schoellmann et al., 2019) have investigated tES on PD patients and have demonstrated it could improve the motor symptoms and modulate the brain oscillatory activity. These findings indicate the potential facilitation effects of tES on stroke recovery and PD symptoms.

### Future Directions

Major knowledge gaps still exist in understanding the mechanism of tES on human motor skills. Currently, the number of studies that have employed neuroimaging techniques is limited. Thus, the interpretation of the effect on motor behavior by tES is limited. Even if behavioral changes are not observed, underlying changes may still result from the stimulation. Thus, coupling with neuroimaging information is essential to advance the understanding of tES and human motor performance. Second, the motor tasks studied in the multimodality studies have largely been basic motor skill tasks, such as simple finger movement or tapping tasks. Complex motor functions have been shown to be enhanced by tES, for example, bimanual coordination tasks (Pixa and Pollok, 2018) or fine motor tasks such as laparoscopic surgical performance (Ciechanski et al., 2017, 2018). However, neuroimaging was not coupled with those complex tasks to understand how they are affected by tES. Last, most of the neuromodulation techniques have poor specificity of intervention. Even though the individual difference exists, the protocol is identical across the population. Other factors such as scalp shape and emotional and physical state are not considered. Neuroimaging techniques could be used to address these limitations by guiding neuromodulation and enabling personalized adaptive stimulation. The combination of neuroimaging and neuromodulation could also form a closed-loop real-time regulatory mechanism (Thut et al., 2017). This could be the next generation of the multimodality technique of neuroimaging and neuromodulation.

## Conclusion

In conclusion, from this review, neuroimaging can be integrated with tES to offer valuable information about how tES affects human motor skills. However, more work utilizing neuroimaging is needed to better understand the underlying mechanism, to advance tES in clinical settings, and to develop the next generation of tES techniques.

## Author Contributions

YG, XI, and SD conceived the original idea. YG designed and performed the review and wrote the manuscript. LC offered essential idea to the review design. LC, XI, and SD edited the manuscript. All authors discussed the conclusions and commented on the manuscript.

## Conflict of Interest

The authors declare that the research was conducted in the absence of any commercial or financial relationships that could be construed as a potential conflict of interest.
